# A Healthcare Finance Model for the United States: A Conceptual Workflow for Episode-Based Pricing, Risk Sharing, and Value-Based Accountability Using Bundled Payment as the Core Mechanism

**DOI:** 10.7759/cureus.112735

**Published:** 2026-07-15

**Authors:** Dominika Zak

**Affiliations:** 1 Analytics, Fordham University, New York, USA

**Keywords:** bundled payment for care improvement, episode-based payment, healthcare finance, us healthcare, value-based care

## Abstract

The United States healthcare system is increasingly moving toward bundled payments as a model for value-based accountability. This approach functions as a healthcare finance model in which the episode of care serves as the central unit of payment design. Rather than reimbursing individual services separately, it organizes costs, risk, incentives, accountability, and quality measurement around the full clinical episode, making the episode the core framework for both financial and care delivery decisions. Under traditional fee-for-service reimbursement, services are paid separately, which can fragment care delivery, reduce cost predictability, and weaken accountability for total episode spending. In contrast, bundled payment models establish episode-level financial accountability by linking benchmark pricing, risk adjustment, care coordination, quality measurement, reconciliation, and payment integrity oversight within a single payment framework. This report develops a conceptual episode-based payment workflow for bundled payments in value-based care. The proposed workflow includes eight stages: episode selection, episode boundary definition, benchmark pricing, risk adjustment, care delivery and tracking, quality measurement, financial reconciliation, and payment integrity monitoring. By framing bundled payments as an integrated finance workflow, this report contributes a structured model for understanding how episode-based reimbursement can bridge healthcare delivery and financial management. It argues that bundled payments may improve cost predictability, reduce fragmentation, strengthen payment integrity, and support value-based accountability when they are designed with fair benchmarks, appropriate risk adjustment, transparent reconciliation, and strong quality safeguards. However, bundled payment models also require careful implementation to avoid underuse of necessary care, patient selection, excessive provider risk, and financial pressure on organizations serving clinically or socially complex populations.

## Introduction

The United States healthcare system has historically relied heavily on fee-for-service reimbursement, in which providers are paid for each separate visit, test, procedure, or service delivered. Although fee-for-service payment supports itemized reimbursement for discrete services, it can also create incentives for service volume rather than value. When each component of care is billed separately, the total cost of a patient’s episode may become difficult to predict, monitor, or manage. This fragmentation creates challenges for patients, payers, providers, and policymakers because spending accountability is distributed across multiple settings and organizations rather than attached to a clearly defined episode of care. Alternative payment models such as bundled payments have emerged in part to address these limitations by promoting accountability for the total cost and quality of care delivered during a defined episode [1-3].

Value-based care emerged in response to these structural limitations by shifting attention from service volume to quality, outcomes, efficiency, and accountability. Within this broader movement, bundled payments are especially important because they create a defined financial unit around an episode of care. Instead of paying separately for hospitalization, physician services, post-acute care, rehabilitation, readmissions, and follow-up care, the bundled payment model establishes accountability for the total cost and quality of the episode. Evidence from bundled payment programs suggests that episode-based reimbursement can improve care coordination and reduce spending in selected clinical settings while maintaining quality outcomes [1,4,5]. Value-based care is the broader healthcare payment and delivery approach that links reimbursement to quality, outcomes, and efficiency rather than service volume alone. Bundled payment is one specific value-based payment model that applies this principle to a defined clinical episode by holding providers accountable for the cost and quality of that episode.

This report presents a healthcare finance model using bundled payments as its backbone. The core contribution of this report is a conceptual workflow that explains how bundled payments function as an integrated financial system. This workflow links episode selection, episode boundary definition, benchmark pricing, risk adjustment, care delivery, quality measurement, reconciliation, and payment integrity monitoring. Framing bundled payments in this way clarifies how episode-based reimbursement can connect healthcare finance, actuarial logic, clinical operations, and value-based accountability. Prior literature often discusses bundled payment in terms of individual program evaluations or isolated design features such as target pricing, quality metrics, or reconciliation. In contrast, the present framework uniquely positions bundled payment as an integrated finance model that links actuarial design, clinical operations, quality safeguards, reconciliation, and oversight within one episode-based structure. This approach is intended to help payers, providers, policymakers, and researchers understand bundled payment not only as a payment reform mechanism but as a coordinated system of financial governance.

The Comprehensive Care for Joint Replacement (CJR) model provides a useful example of episode-based reimbursement in practice. CMS designed the CJR model to improve quality and cost efficiency for Medicare beneficiaries undergoing lower extremity joint replacement procedures, while holding participating hospitals accountable for episode spending and outcomes [3,4]. In practice, quality in bundled-payment programs is typically assessed using a combination of utilization-based, clinical, and patient-centered measures, depending on the episode and payer. Evaluations of the model have demonstrated reductions in episode spending and post-acute care utilization without clear evidence of harm to quality, supporting bundled payments as a viable approach to healthcare payment reform [2,5].

## Technical report

Bundled payments as a healthcare finance model

Bundled payments offer an alternative to fragmented fee-for-service reimbursement by grouping services related to a defined clinical episode into one payment framework. The financial logic of bundled payments is based on episode-level accountability. A payer or contracting entity establishes a benchmark or target price for an episode of care. Providers may continue to submit claims during the episode, but total spending is later compared with the benchmark. If actual spending is below the target and quality requirements are met, providers may receive shared savings or a reconciliation payment. If spending exceeds the benchmark, providers may be responsible for repayment in models that include downside risk.

The CJR model illustrates this financial structure. In this model, participating hospitals are financially accountable for the quality and cost of a joint replacement episode, including care during and after hospitalization. CMS has also reported that the CJR model generated savings for Medicare while maintaining quality for large numbers of hip and knee replacement patients [4]. These features make bundled payments more than a reimbursement method. They function as a financial governance structure that connects pricing, clinical coordination, quality measurement, and accountability.

Bundled payments occupy an important middle position between fee-for-service and global payment. Fee-for-service creates accountability for individual services but weak accountability for the total cost of care. Global payment creates accountability for a broad population but may be too broad for organizations that are not prepared to manage full population risk. Bundled payment creates a focused financial unit around a specific episode, making it possible to measure cost, utilization, quality, and outcomes within a clinically meaningful time frame. This structure is particularly useful for episodes with clear start points, predictable care pathways, and measurable outcomes.

Evidence supporting bundled payment models

The evidence on bundled payments suggests that these models can reduce spending in selected clinical areas, particularly surgical episodes. A systematic review of CMS bundled payment programs found that bundled payment models were generally associated with spending reductions for surgical episodes, especially orthopedic procedures, while findings for medical episodes were more mixed [1]. This distinction is important because surgical episodes often have clearer boundaries, more predictable service patterns, and more measurable post-acute care utilization than chronic or complex medical conditions.

The CJR model is one of the most studied examples of mandatory bundled payment. Evaluation of the first two years of mandatory bundled payments for hip and knee replacement demonstrated modest reductions in episode spending without an increase in complication rates [2]. The CJR model was also associated with reduced Medicare Part A spending, largely through reductions in post-acute care spending [5]. These findings suggest that bundled payments may reduce avoidable spending by encouraging hospitals, physicians, and post-acute providers to coordinate discharge planning, rehabilitation, follow-up care, and readmission prevention.

Bundled payments have also been highlighted as an important component of the broader shift from fee-for-service reimbursement to value-based health insurance [6-8]. Episode-based accountability may support the transition toward value-based insurance design by creating financial incentives that reward coordination, quality, and efficient resource utilization.

However, the literature does not support the conclusion that bundled payments automatically succeed across all clinical settings. Bundled payments for selected medical conditions did not produce the same clear savings observed in some surgical bundles [6]. Medical episodes may be more difficult to bundle because they often involve greater clinical uncertainty, multiple comorbidities, longer time horizons, and stronger influence from social risk factors. Therefore, bundled payment design must include careful episode selection, strong risk adjustment, quality safeguards, and protections for unusually complex cases.

Conceptual approach

This report uses a conceptual policy and finance framework rather than original empirical analysis. The purpose is to synthesize existing literature and develop a structured workflow for understanding bundled payments as a healthcare finance model. The model is informed by evidence from bundled payment evaluations, policy reports, and prior work on value-based care, payment integrity, and alternative payment models [7-9]. Conceptual frameworks are useful when a field has multiple empirical findings but lacks a clear operational model connecting those findings to implementation. In this case, the proposed workflow organizes the financial mechanics of bundled payment into a sequence that can be used by payers, providers, policymakers, and researchers to evaluate episode-based reimbursement design.

Proposed episode-based payment workflow

The proposed workflow begins with episode selection. Payers and providers should select episodes that are clinically definable, high cost, high volume, measurable, and associated with opportunities for coordination or efficiency improvement. Examples may include joint replacement, cardiac procedures, maternity care, selected cancer surgeries, and certain high-cost hospital episodes. Episode selection is a financial design decision because the wrong episode may shift risk to providers without creating a realistic opportunity to improve value.

The next stage is episode boundary definition. This requires defining the clinical trigger, included services, participating providers, time window, exclusions, and accountability structure. For example, a surgical bundle may begin with hospital admission and continue for 30 or 90 days after discharge. It may include the index hospitalization, professional fees, post-acute care, rehabilitation, complications, readmissions, and follow-up visits. Episode-level structures can improve transparency by making cost and utilization easier to observe across the full care continuum instead of across disconnected claims [7].

After the episode boundary is defined, the model requires benchmark pricing. The benchmark price should reflect historical spending, regional and national spending patterns, expected service use, and quality expectations. Benchmark pricing is the financial foundation of the bundle because it determines whether providers are being held to a realistic standard. If the benchmark is too high, the model may not generate meaningful savings. If the benchmark is too low, providers may face unsustainable financial pressure.

Risk adjustment is the next stage and is essential to fairness. Providers caring for older, sicker, or socially complex patients should not be penalized simply because their patients require more resources. Weak risk adjustment may create incentives for providers to avoid high-risk patients or reduce access for populations with complex needs. A fair bundled payment model should also include outlier protection, stop-loss arrangements, or other safeguards for cases that fall outside expected clinical variation.

The care delivery and tracking stage is where the finance model becomes operational. Providers must manage discharge planning, post-acute referrals, rehabilitation, medication management, complications, and follow-up care within the structure of the episode. This does not mean withholding necessary care. Instead, it means reducing duplicative services, avoidable variation, unnecessary institutional post-acute care, preventable complications, and readmissions. Reductions in post-acute care spending have been identified as an important driver of savings in joint replacement bundles [5], suggesting that bundled payments can influence how organizations manage transitions across care settings.

Quality measurement must be integrated into the financial model. Bundled payments could create incentives to reduce services too aggressively if quality safeguards are weak. For this reason, quality measures should include readmissions, complications, mortality, patient-reported outcomes, functional improvement, patient experience, and equity-sensitive measures. Savings should not be rewarded if they are achieved through unsafe discharge, reduced access, or poor outcomes. Quality measurement protects patients and helps distinguish true efficiency from cost shifting or underuse.

Financial reconciliation is the closing stage of the bundled payment cycle. At the end of the episode, actual spending is compared with the benchmark price. If actual spending is below the benchmark and quality requirements are met, providers may receive shared savings or a reconciliation payment. If spending is above the benchmark, providers may repay part of the loss if the model includes downside risk. Reconciliation transforms a bundled payment into a performance-based financial contract because payment depends on the relationship between the benchmark price, actual spending, and quality performance.

Payment integrity monitoring is the final stage of the workflow. Bundled payments can strengthen payment integrity by making abnormal utilization patterns more visible [7]. Under fee-for-service, inappropriate utilization may be hidden within many separate claims. Under bundled payment, analysts can compare total episode cost, service mix, post-acute utilization, readmissions, and outcomes against expected benchmarks. This structure does not eliminate fraud, abuse, coding inflation, or inappropriate utilization, but it creates a more transparent basis for identifying unexplained variation. Payment integrity, therefore, shifts from a mostly retrospective audit function toward a more proactive finance and governance function.

**Figure 1:**
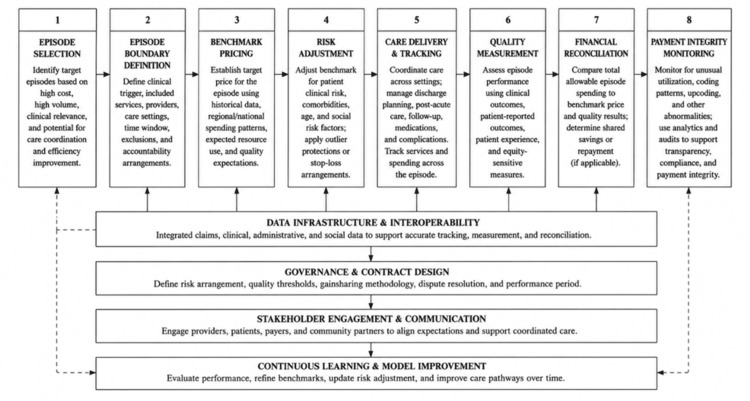
Proposed Episode-Based Payment Workflow for Bundled Payments The workflow illustrates eight sequential stages in an episode-based bundled payment model: episode selection, episode boundary definition, benchmark pricing, risk adjustment, care delivery and tracking, quality measurement, financial reconciliation, and payment integrity monitoring. The model aligns these stages with value-based objectives, including cost predictability, fairness, care coordination, quality safeguards, and accountability.

Figure 1 shows the proposed episode-based payment workflow for bundled payments.

## Discussion

Bundled payments should be understood as a healthcare finance model because they redesign how money flows through the care system. The central financial question changes from “How many services were provided?” to “Was the full episode delivered efficiently, safely, and within an appropriate cost range?” This shift is central to value-based care because it aligns financial incentives with coordination, accountability, and outcomes [7-9]. It also creates stronger cost predictability for payers and clearer accountability for providers.

The proposed workflow contributes to the literature by translating bundled payment from a policy concept into an operational finance model. The workflow shows that a bundled payment is not a single transaction. It is a structured system that links pricing, risk, care management, quality, reconciliation, and oversight. This framing may be useful for healthcare finance leaders because it identifies the core design elements that must be present for bundled payments to function responsibly. It may also be useful for policymakers because it clarifies where bundled payment models can fail if risk adjustment, quality safeguards, or reconciliation methods are poorly designed. Recent work has also emphasized the importance of episode-level transparency and technology-enabled infrastructure to support accountability across bundled payment arrangements [10]. This eight-stage workflow is a conceptual finance and policy framework synthesized from prior literature and policy evaluations, intended to organize the operational components of bundled payment design rather than to claim demonstrated implementation success.

The model also highlights the importance of financial fairness. Bundled payment can create strong incentives for efficiency, but it can also transfer risk to providers. If providers lack data infrastructure, actuarial support, care coordination capacity, or financial reserves, bundled payments may become difficult to manage. Smaller hospitals, rural providers, and safety-net organizations may face greater challenges if they serve patients with more complex clinical and social needs. Therefore, policymakers should consider phased implementation, technical assistance, risk corridors, stop-loss protection, and equity-sensitive risk adjustment.

Another important implication is that bundled payment should not be evaluated only by whether it reduces spending. A successful bundled payment model should reduce unnecessary spending while preserving or improving quality. It should also improve transparency, strengthen accountability, and reduce fragmentation across the care continuum [1,2,5]. Spending reductions that result from avoiding needed care, excluding complex patients, or shifting costs to other settings should not be considered true value. For this reason, bundled payment contracts must incorporate both financial and clinical safeguards.

The evidence on bundled payments suggests that these models can reduce spending in selected clinical settings, but the strength and consistency of the evidence vary substantially by episode type. The most consistent evidence comes from surgical episodes, particularly orthopedic procedures, where episode boundaries are relatively clear, care pathways are more standardized, and post-acute utilization is easier to measure and manage. In contrast, medical episodes are more heterogeneous and need more empirical studies to establish more robust data for proof of concept.

Limitations and future recommendations

This technical report is conceptual and does not present original empirical analysis. The proposed workflow is based on existing literature and policy evidence, but it has not been tested as a formal implementation model. Future research should evaluate whether organizations that adopt this type of integrated workflow achieve better financial, quality, and payment integrity outcomes than organizations using less structured bundled payment designs. Additional research is also needed to examine how bundled payment models affect safety-net providers, rural hospitals, medically complex patients, and populations with high social risk. These questions are important because value-based payment reforms may produce uneven effects across healthcare settings.

## Conclusions

Bundled payments represent an important healthcare finance model for value-based care because they convert fragmented service-level billing into episode-level financial accountability. By defining the episode, setting a benchmark price, adjusting for risk, monitoring care delivery, measuring quality, reconciling performance, and tracking payment integrity, bundled payments create a structured workflow for aligning cost and outcomes. Evidence from surgical bundles, especially joint replacement, suggests that bundled payments can reduce spending without clear harm to quality when the model is well designed. However, results are more mixed for medical episodes, which show the importance of careful episode selection and fair financial design. The proposed episode-based payment workflow provides a practical framework for understanding bundled payments not only as payment reform but as a complete finance system for value-based healthcare.

## References

[1] Agarwal R, Liao JM, Gupta A, Navathe AS (2020). The impact of bundled payment on health care spending, utilization, and quality: a systematic review. Health Aff (Millwood).

[2] Kodan A (2026). Enhancing payment integrity in U.S. healthcare through value-based care: the role of bundled episodes and alternative payment models. Cureus.

[3] Barnett ML, Wilcock A, McWilliams JM (2019). Two-year evaluation of mandatory bundled payments for joint replacement. N Engl J Med.

[4] (2026). Centers for Medicare & Medicaid Services: Comprehensive care for joint replacement model. 2026.

[5] (2026). Centers for Medicare & Medicaid Services: Innovation insight: comprehensive care for joint replacement (CJR) model generates savings to Medicare. 2026.

[6] Haas DA, Zhang X, Kaplan RS, Song Z (2019). Evaluation of economic and clinical outcomes under Centers for Medicare & Medicaid Services mandatory bundled payments for joint replacements. JAMA Intern Med.

[7] Joynt Maddox KE, Orav EJ, Zheng J, Epstein AM (2018). Evaluation of Medicare’s bundled payments initiative for medical conditions. N Engl J Med.

[8] Kodan A (2026). Artificial intelligence as a catalyst for value-based health insurance in the United States: narrative review and policy perspective. JMIR AI.

[9] Mechanic RE, Altman SH (2009). Payment reform options: episode payment is a good place to start. Health Aff (Millwood).

[10] Kodan A (2026). Enabling episode-level transparency in value-based care through large language model-driven provider directories. Cureus.

